# Contribution of Brain Size to IQ and Educational Underperformance in Extremely Preterm Adolescents

**DOI:** 10.1371/journal.pone.0077475

**Published:** 2013-10-09

**Authors:** Jeanie L. Y. Cheong, Peter J. Anderson, Gehan Roberts, Alice C. Burnett, Katherine J. Lee, Deanne K. Thompson, Carly Molloy, Michelle Wilson-Ching, Alan Connelly, Marc L. Seal, Stephen J. Wood, Lex W. Doyle

**Affiliations:** 1 Neonatal Services, Royal Women’s Hospital, Melbourne, Australia; 2 Victorian Infant Brain Studies, Murdoch Childrens Research Institute, Melbourne, Australia; 3 Department of Obstetrics & Gynaecology, University of Melbourne, Melbourne, Australia; 4 School of Psychological Sciences, University of Melbourne, Melbourne, Australia; 5 Royal Children’s Hospital, Melbourne, Australia; 6 Department of Paediatrics, University of Melbourne, Melbourne, Australia; 7 Developmental Imaging, Murdoch Childrens Research Institute, Melbourne, Australia; 8 Florey Institute of Neurosciences and Mental Health, Melbourne, Australia; 9 School of Psychology, University of Birmingham, Edgbaston, United Kingdom; University Children's Hospital Tuebingen, Germany

## Abstract

**Objectives:**

Extremely preterm (EP) survivors have smaller brains, lower IQ, and worse educational achievement than their term-born peers. The contribution of smaller brain size to the IQ and educational disadvantages of EP is unknown. This study aimed (i) to compare brain volumes from multiple brain tissues and structures between EP-born (<28weeks) and term-born (≥37weeks) control adolescents, (ii) to explore the relationships of brain tissue volumes with IQ and basic educational skills and whether this differed by group, and (iii) to explore how much total brain tissue volume explains the underperformance of EP adolescents compared with controls.

**Methods:**

Longitudinal cohort study of 148 EP and 132 term controls born in Victoria, Australia in 1991-92. At age 18, magnetic resonance imaging-determined brain volumes of multiple tissues and structures were calculated. IQ and educational skills were measured using the Wechsler Abbreviated Scale of Intelligence (WASI) and the Wide Range Achievement Test(WRAT-4), respectively.

**Results:**

Brain volumes were smaller in EP adolescents compared with controls (mean difference [95% confidence interval] of -5.9% [-8.0, -3.7%] for total brain tissue volume). The largest relative differences were noted in the thalamus and hippocampus. The EP group had lower IQs(-11.9 [-15.4, -8.5]), spelling(-8.0 [-11.5, -4.6]), math computation(-10.3 [-13.7, -6.9]) and word reading(-5.6 [-8.8, -2.4]) scores than controls; all p-values<0.001. Volumes of total brain tissue and other brain tissues and structures correlated positively with IQ and educational skills, a relationship that was similar for both the EP and controls. Total brain tissue volume explained between 20-40% of the IQ and educational outcome differences between EP and controls.

**Conclusions:**

EP adolescents had smaller brain volumes, lower IQs and poorer educational performance than controls. Brain volumes of multiple tissues and structures are related to IQ and educational outcomes. Smaller total brain tissue volume is an important contributor to the cognitive and educational underperformance of adolescents born EP.

## Introduction

Preterm birth is associated with altered brain growth and development [1]. Cerebral white matter injury has long been recognised as the primary brain lesion affecting very preterm infants [1]. However, there is increasing evidence of gray matter involvement due to secondary neuronal and axonal injury, prompting the term “encephalopathy of prematurity” to encompass pathology involving both white and gray matter [2,3]. Brain tissues and structures that have been implicated include the cortical gray matter, thalamus, basal ganglia, hippocampus, amygdala and corpus callosum [4,5].

Functional deficits in cognitive and educational domains in mid-childhood and adolescence have been reported in preterm cohorts worldwide [6]. In a comparative study of 4 countries, extremely low birthweight children were assessed between the ages of 8 to 11 years, and the proportion of the extremely low birthweight children who performed within the normal range for IQ and measures of educational skills were between 40 to 80%, with more than half the cohort requiring special educational assistance or repeating a grade at school [7].

Magnetic resonance imaging (MRI) studies in humans have enabled a better understanding of the consequences of preterm birth on brain development as well as brain structure-function relationships. A recent meta-analysis of brain volumes in very preterm (<32 weeks’ gestation) or very low birthweight (<1500 g) children and adolescents reported smaller brain volumes in white matter, gray matter, corpus callosum, hippocampus and cerebellum compared with term-born controls [8]. Due to insufficient data, conclusions could not be drawn about other important brain structures potentially vulnerable to preterm birth such as the thalamus, basal ganglia or amygdala in that meta-analysis; although there are individual studies that report smaller volumes of the thalamus [9,10], basal ganglia [4], caudate nucleus [9,11] and amygdala [4,9,11]. In this meta-analysis, smaller brain volumes were associated with reduced general intelligence (IQ) as well as poorer language, memory and executive functioning skills for preterm children at ages 8 to 18 years [8], but whether this relationship also holds for basic educational skills is not known. Moreover, how much brain volume contributes to differences in cognitive and educational outcomes between preterm and term controls in childhood and adolescence has not been described. It is important to note other limitations of the studies included in the meta-analysis that may affect the generalizability of the conclusions. Firstly, most studies reported MRI alterations in adolescents born before the 1990s, before exogenous surfactant was available, when survival rates were lower than subsequently. In addition, sample sizes in the component studies were small and from non-geographic cohorts. Thus, the aim of the current study was to address these research gaps by using a large geographically-representative neuroimaging cohort of extremely preterm (EP, <28 weeks’ gestational age at birth) born when surfactant was available, to determine the importance of volumes of different brain tissues and structures in relation to critical functional outcomes of IQ and basic educational skills in EP and term-born adolescents.

Specifically, this study aimed to:

1 Compare volumes of various brain tissues and structures between EP and term-born (gestational age ≥37 weeks) control adolescents.
2 Explore the relationships between total brain tissue volume, and IQ and basic educational skills, and whether this differed between EP and term-born adolescents.
3 To explore how much total brain tissue volume explains the lower performance of EP adolescents compared with controls on measures of IQ and basic educational skills.

We hypothesized that EP adolescents would have smaller volumes of total brain tissue, cortical gray matter, white matter, thalamus, basal ganglia, cerebellum, hippocampus and amygdala compared with controls. We expected that larger total brain tissue and other brain volumes would be associated with better performance on measures of IQ and basic educational skills in adolescence, and that this relationship would be similar for EP and control adolescents. We also hypothesized that smaller total brain tissue volume would at least partially explain the lower scores on measures of IQ and basic educational skills in the EP adolescents compared with controls.

## Materials and Methods

### Participants

This study comprised a geographically-representative, prospective cohort of all EP survivors born in the state of Victoria, Australia, between January 1991 and December 1992. Controls were healthy term-born infants contemporaneously recruited from each of the three tertiary perinatal hospitals in the state, matched to the EP cohort on expected date of birth, mother’s country of birth (English-speaking versus other) and health insurance status (private or public). Study participants had been assessed previously at ages 2, 5 and 8 years [12-15]. At age 18 years, the participants were invited to attend a comprehensive health and developmental follow-up including an MRI scan.

Ethical approval for the original and follow-up studies was obtained from the Human Research Ethics Committees of the Royal Women’s Hospital, Mercy Hospital for Women, Monash Medical Centre and Royal Children’s Hospital, Melbourne. Written informed consent was obtained from all participants, including their parents if they were younger than 18 years of age at the time of assessment.

### MRI

MRI was performed at two of the study sites, each using a Siemens 3T MAGNETOM Trio MRI system (Siemens, Erlangen, Germany), a 12 channel receive-only head coil, and the same acquisition protocol. For this study, 3-dimensional T1-weighted MPRAGE (Magnetisation Prepared Rapid Gradient Echo) datasets were obtained using the following parameters: non-isotropic 0.7 x 0.7 x 1.2 mm, field of view 230 mm, repetition time 1800 msec, echo time 2.67 msec and flip angle 9°.

### Image analysis

Data were processed by a single operator, blinded to group status, on Linux workstations using the automated FreeSurfer image processing suite (stable release version 5.0, http://surfer.nmr.mgh.harvard.edu) [16,17]. Images were visually inspected for artifacts and abnormalities that could compromise automated processing. Images that were considered of unacceptably poor quality due to artifact or major cerebral structural abnormalities that affected registration were excluded (EP n=14, control n=10). During the automated image processing pipeline, the MR images were visually inspected and manually edited as required.

The automated labeling system for whole brain segmentation within FreeSurfer was used and the volumes of the following tissues and structures were estimated; cortical gray matter, white matter, thalamus, caudate, putamen, pallidum, hippocampus, amygdala, cerebellar white and gray matter, and intracranial volume (including the ventricular volumes) [18,19]. With the exception of intracranial volume, the volume for each brain tissue or structure was estimated separately for each hemisphere. For the purposes of this study, we combined volumes from both hemispheres for each brain tissue or structure. The total volumes for caudate, putamen and pallidum were combined to represent basal ganglia volume. Cerebellar volume was taken as the sum of cerebellar white and gray matter. Total brain tissue volume was the combined volumes of all the above brain structures including the brainstem (i.e. excluding cerebrospinal fluid volume).

### Functional outcomes

IQ was estimated using a two-subtest version of the Wechsler Abbreviated Scale of Intelligence (WASI) [20]. The WASI has excellent psychometric properties and the two-subtest full-scale IQ correlates highly (r >0.80) with the equivalent full-scale IQ scores for the age appropriate Wechsler Intelligence Scale for Children (WISC-III) [21] and Wechsler Adult Intelligence Scale (WAIS-III) [22]. IQ is a standardized scale with a mean of 100 and standard deviation (SD) of 15.

Basic educational skills of word reading (single word decoding), spelling, and math computation were assessed using the relevant subtests from the Wide Range Achievement Test (WRAT4) [23]. The WRAT4 also has excellent psychometric properties and is used extensively in clinical and research settings. All scales are age standardized with a mean of 100 and SD of 15.

### Statistical analysis

Data were analyzed using STATA 12.0 (StatCorp, Texas, USA). Participant characteristics were compared (i) between EP and control groups, as well as (ii) participants with and without analyzable MRI within the EP and control groups, using t-tests or chi-square analyses. Group differences in brain volumes were explored using linear regression fitted using generalized estimating equations with robust (sandwich) estimation of standard errors to allow for multiple births within a family, using separate models for each brain tissue or structure. The regression was performed first unadjusted, and then adjusted for sex and intracranial volume. All analyses were adjusted for age at the time of the MRI and for study site of scan to account for this potential confounder. Results are presented as the mean volumetric difference between the EP and control groups and 95% confidence interval (CI) in cubic centimeters (cc), as well as the percentage difference calculated as the volumetric difference divided by the mean size of the brain tissue or structure in the controls.

The relationships between different brain volumes and functional outcomes were explored using linear regression again fitted using generalized estimating equations with robust (sandwich) estimation of standard errors, with a separate model for each volume-outcome combination. The analysis was repeated adjusting for sex, maternal education and social class; recognizing maternal education and social class to be important factors affecting cognitive and academic outcomes [24,25]. Maternal education was dichotomized into less than or greater than/equal to 12 years of schooling. Social class was based on the occupation of the major income earner in the family and was categorized as lower (unskilled or unemployed) or higher (semi-skilled, skilled or professional). Both maternal education and social class data were collected at the 8 year follow up. We then assessed whether the relationship between brain volume and functional outcomes was similar in EP and control adolescents by allowing an interaction between brain volume and group.

The differences in functional outcomes between groups were modelled using linear regression fitted using generalized estimating equations with robust (sandwich) estimation of standard errors, with and without adjustment for sex and sociodemographic variables. To explore how much total brain tissue volume explains the differences in IQ and educational outcomes between groups, total brain tissue volume was added to the adjusted regression analyses and the % change in the estimated mean difference between EP and control groups and the relative increase in the variance in outcome explained by the model (measured by R-squared) were calculated relative to the unadjusted model.

Given the multiple comparisons, we have interpreted our findings by focusing on overall patterns and magnitude of differences, rather than on individual p-values.

## Results

Of the 225 EP and 253 control subjects known to be alive at 8 years, 176 (78%) EP and 155 (61%) controls were seen as part of the follow-up study at 18 years. One hundred and sixty-two EP and 143 control participants (92% of those who attended the 18-year follow up for both groups) consented to an MRI examination, of which 148 and 132 scans, respectively, were suitable for FreeSurfer analysis; these subjects formed the study groups.

The proportion of males and small for gestational age at birth in the EP and control groups was similar, but there were expected differences in all other characteristics (Table 1). Compared with EP subjects who were not included in this study (i.e. those without analyzable MRI data and those not assessed at 18 years of age), EP participants included in this study had lower rates of cystic periventricular leukomalacia in the newborn period (4.1% vs 13.0%) and cerebral palsy at 8 years (7.6% vs 24.2%), and higher full scale IQ at 8 years (mean difference [95% CI] of 6.2 [1.1, 11.2]). Controls who were included in this study were more likely to be female (58.3% vs 45.4%) and had higher full scale IQ at 8 years (mean difference [95% CI] of 7.3 [3.5 to 11.1]) than controls not included in this study; other characteristics were similar.

**Table 1:** Participant characteristics in EP adolescents compared with controls.

	**EP (n=148)**	**Controls (n=132)**
Antenatal corticosteroids	102 (68.9)	0 (0)^
Maternal schooling less than 12 years	74 (51.8)^$^	41 (32.0)^@^
Lower social class	47 (32.9)^$^	23 (17.8)^&^
Multiple births	35 (23.6)	0 (0)
Gestational age at birth in weeks - mean (SD)	25.8 (1.1)	39.3 (1.3)
Birthweight in grams - mean (SD)	897 (177)	3441 (457)
Birthweight <-2 SD	4 (2.7)	0 (0)
Male	70 (47.3)	55 (41.7)
Intraventricular hemorrhage grade 3 or 4	10 (6.8)	0 (0)
Cystic periventricular leukomalacia	6 (4.1)	0 (0)
Bronchopulmonary dysplasia	57 (38.5)	0 (0)
Postnatal corticosteroids	55 (37.2)	0 (0)
Retinopathy of prematurity stage 3 or worse	20 (13.7)^%^	0 (0)
Cerebral palsy*	11 (7.6)^&^	0 (0)
IQ <70	6 (4.1)^	0 (0)^
Blindness+	0 (0)	0 (0)
Hearing impairment requiring hearing aids	1 (0.7%)^	0 (0)
Received educational assistance	31 (23.7)^@^	12 (9.4)^&^
Repeated a grade at school	25 (17.6)**	8 (6.3)^@^

Data are number (%) unless otherwise specified. EP = extremely preterm. SD = standard deviation. * Cerebral palsy confirmed at age 8 years. ^+^ Visual acuity <20/200 in the better eye. Data unavailable for: ^n=1, ^%^n=2, ^&^n=3, ^@^n=4, ^$^n=5, ^**^n=6 participants

There was evidence that EP participants had smaller volumes for all brain tissues and structures compared with controls (Table 2), ranging from a mean difference of -5.9% for total brain tissue volume, up to -10.7% for the thalamus. After adjustment for intracranial volume and sex, the evidence for differences remained in all brain regions, although was somewhat weaker for cortical gray and cortical white matter.

**Table 2:** Brain volumes in EP adolescents compared with controls.

**Brain tissue or structure**	**Brain volume (cc); Mean (SD)**		**Mean difference (95% CI)**		**P value**	Adjusted mean difference* (95% CI)		**P value**
	**EP (n=148)**	**Controls (n=132)**	**Volume (cc)**	**Volume (%)^&^**		**Volume (cc)**	**Volume (%)^&^**	
Total brain tissue	1289.8 (128.0)	1371.6 (126.4)	-80.3 (-110.3, -50.4)	-5.9 (-8.0, -3.7)	<0.001	-89.1 (-114.1, -64.1)	-6.5 (-8.3, -4.7)	<0.001
Cortical grey matter	498.1 (49.3)	523.5 (49.0)	-24.4 (-35.9, -12.9)	-4.7 (-6.9, -2.5)	<0.001	-6.9 (-12.5, -1.3)	-1.3 (-2.4, -0.3)	0.02
Cortical white matter	438.1 (54.3)	466.0 (52.3)	-27.9 (-40.8, -15.0)	-6.0 (-8.8, -3.2)	<0.001	-6.8 (-14.0, 0.4)	-1.5 (-3.0, 0.1)	0.06
Thalamus	14.2 (1.6)	15.9 (1.6)	-1.7 (-2.1, -1.3)	-10.7 (-13.2, -8.2)	<0.001	-1.1 (-1.4, -0.8)	-6.9 (-8.8, -5.0)	<0.001
Basal ganglia	22.0 (2.6)	23.6 (2.5)	-1.6 (-2.2, -1.0)	-6.8 (-9.3, -4.2)	<0.001	-0.8 (-1.2, -0.3)	-3.4 (-5.1, -1.3)	0.001
Cerebellum	143.3 (17.7)	154.0 (15.7)	-10.5 (-14.4, -6.6)	-6.8 (-9.4, -4.3)	<0.001	-5.9 (-8.5, -3.3)	-3.8 (-5.5, -2.1)	<0.001
Hippocampus	8.1 (0.8)	8.8 (0.8)	-0.7 (-0.9, -0.5)	-8.0 (-10.2, -5.7)	<0.001	-0.5 (-0.7, -0.4)	-5.7 (-8.0, -4.6)	<0.001
Amygdala	3.3 (0.3)	3.5 (0.4)	-0.2 (-0.3, -0.1)	-5.7 (-8.6, -2.9)	<0.001	-0.1 (-0.2, -0.05)	-2.9 (-5.7, -1.4)	0.001

All analyses were adjusted for age at MRI; *also adjusted for intracranial volume and sex (with the exception of total brain volume, which was only adjusted for sex); ^&^ Volume difference expressed as a percentage of the mean volume in the controls for the particular brain tissue or structure. EP = extremely preterm. CI = confidence interval. cc = cubic centimeters. SD = standard deviation.

**Figure 1:**
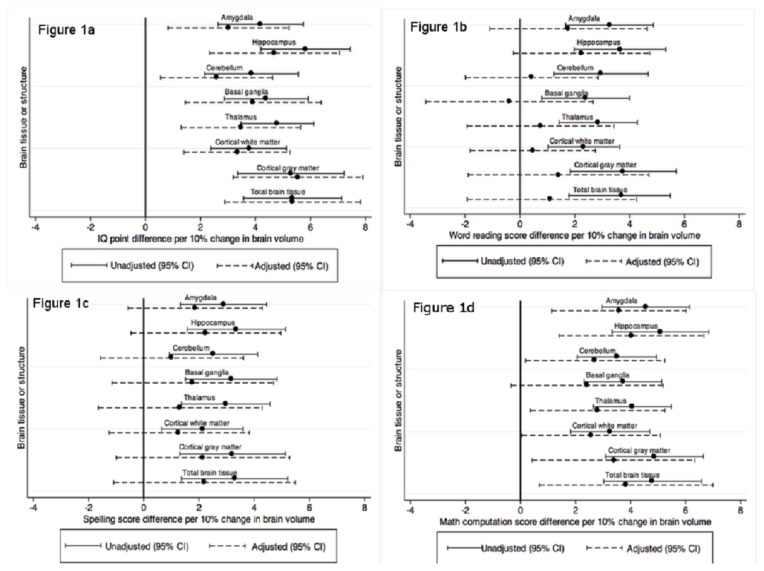
Relationships between brain tissue volumes and IQ/educational outcomes. Relationships between brain volumes of multiple tissues and structures with IQ (Figure 1a), word reading (Figure 1b), spelling (Figure 1c) and math computation (Figure 1d) scores, with data from extremely preterm and control subjects combined. The solid lines represent unadjusted analyses; the dashed lines represent analyses adjusted for sex, maternal education and social class. The x-axis shows score difference for every 10% difference in brain volume, relative to the volume of the particular brain region in controls. CI – confidence interval.

Full scale IQ increased with increasing volumes for all tissues and structures for the entire cohort (Figure 1a), a relationship which remained after adjustment for sex, maternal education and social class. For word reading, spelling, and math computation, there was evidence that all scores increased with increasing brain volumes in the unadjusted analysis (Figure 1b, 1c, 1d). Following adjustment for sex, maternal education and social class, effect sizes were reduced and became non-significant for word reading and spelling (Figure 1b and 1c), however the evidence for relationships for all brain volumes and math computation remained after adjustment although effect sizes were again reduced (Figure 1d). There was little evidence that the relationship between brain volumes and IQ or measures of basic educational skills varied between the EP and control groups (all interactions, p>0.05).

EP adolescents had lower IQs and worse academic performance in all areas compared with controls (Table 3). The evidence for differences remained after adjustment for sociodemographic variables (sex, social class and maternal education), but when adjusted simultaneously for total brain tissue volume and sociodemographic variables, the evidence for a difference in reading scores between groups reduced. Total brain tissue volume explained between 20-40% of the IQ and educational outcome differences between EP and control adolescents, based on the reduction in the mean differences between the groups when total brain tissue volume was added to the models adjusted for sociodemographic variables (Table 3).

**Table 3:** IQ and academic performance at 18 years of age in EP adolescents compared with controls, and effects of total brain tissue volume on the relationships between groups.

**Outcome**	**Mean score (SD)**		**Mean difference (95% CI); P-value; r^2^**			**% reduction in mean difference#**	Relative increase in r^2^*
	**EP (n=147)**	**Controls (n=131)**	**Unadjusted**	**Adjusted**†	**Adjusted**‡		
IQ	95.7 (15.9)	107.6 (12.8)	-11.9 (-15.4, -8.5); <0.001; 14.5%	-9.1 (-12.4, -5.8); <0.001; 28.2%	-6.2 (-9.6, -2.7); <0.001; 33.1%	31.9%	17.4%
Reading^$^	95.5 (13.5)	101.1 (13.6)	-5.6 (-8.8, -2.4); 0.001; 4.1%	-3.7 (-6.8, -0.6); 0.019; 14.3%	-2.2 (-5.3, 1.0); 0.17; 16.1%	40.5%	12.6%
Spelling	97.1 (15.2)	105.1 (14.0)	-8.0 (-11.5, -4.6); <0.001; 7.1%	-6.0 (-9.5, -2.6); 0.001; 16.0%	-4.0 (-7.2, -0.9); 0.012; 18.4%	33.3%	15.0%
Math computation	85.2 (14.0)	95.6 (14.3)	-10.3 (-13.7, -6.9); <0.001; 11.8%	-8.8 (-12.1, -5.6); <0.001; 24.6%	-7.0 (-10.3, -3.8); <0.001; 26.5%	20.4%	7.7%

^$^Data available for 146 EP; † adjusted for sex, social class and maternal education; ‡ adjusted for total brain tissue volume, sex, social class and maternal education; # percentage reduction in mean difference between groups when total brain tissue volume was added to the model, calculated as 1 – (the mean difference adjusted for sex, social class and maternal education divided by the mean difference adjusted for total brain tissue volume, sex, social class and maternal education); * percentage increase in r^2^ when total brain tissue volume was added to the model, calculated as (r^2^ in the model adjusted for total brain tissue volume, sex, social class and maternal education minus r^2^ in the model adjusted for sex, social class and maternal education) divided by r^2^ in the model adjusted for sex, social class and maternal education. All P-values are for differences between EP and Control groups.

EP = extremely preterm; **r^2^** = % variance explained by the variables in the model; CI = confidence interval; SD = standard deviation.

## Discussion

These results from the largest regional neuroimaging cohort of adolescents born in the 1990s have re-emphasized the findings that EP birth is associated with smaller volumes in multiple brain tissues and structures in adolescence compared with controls. After adjustment for intracranial volume, all brain tissues and structures of interest were smaller in the EP group. Not only were smaller brain volumes associated with lower IQ, but this relationship was also apparent for basic educational skills. The relationship between brain volumes and basic educational skills is a novel finding for both preterm and control adolescents. We also found evidence of an association between amygdala volumes and IQ, which has not been described previously. As expected, EP adolescents had lower scores for IQ and educational performance than controls. Interestingly, total brain tissue volume explained between 20% and 40% of the difference in IQ and educational outcomes between the EP and term adolescents.

The findings of smaller brain volumes in EP compared with term-born adolescents are in keeping with studies involving small sample sizes from earlier eras. Gray and white matter volume differences between very preterm (<33 weeks’ gestation) and term controls at ages ranging from 8 to 17 years have been reported to be in the order of 5-12%, and 2.5-9% respectively [9,10,26-28], although not all studies reported evidence of group differences after adjustment for intracranial volume [10,27]. Despite being a cohort who were more preterm at birth, the current study found reductions of gray and white matter of similar magnitude to those reported in previous studies.

The magnitudes of differences of around 6% for the total brain volume and 8% for the hippocampus are similar to several previous reports [9,10,27,29], with the exception of one study where a 12-16% reduction of hippocampal volume was found [27]. The cohort in the latter study was more mature (<33 weeks’ gestation) than in the current study, was born in the late 1970s, and manual tracing was used to determine hippocampal volumes. Studies comparing manual and automated segmentation of the hippocampus suggest that manual hippocampal volume estimation often results in smaller volumes than automated techniques [30,31]. However, one study comparing the Freesurfer software suite and manual tracing of the hippocampus found good reliability and validity of the automated method, and supported the use of Freesurfer in the context of large samples where group differences rather than absolute volumes were the focus of the study [31].

We are able to confirm findings of smaller thalami, basal ganglia, amygdala and cerebellum in our EP cohort compared with term-born controls, which is a similar finding to that in more mature cohorts [9-11]. The largest relative reduction was found in the thalamus, which further supports the increasing evidence highlighting the thalamus as one of the more vulnerable structures affected by preterm birth [32], although we note that there were large overlaps in the confidence intervals across the different tissues and structures.

Brain volumes have been associated with a range of cognitive outcomes in childhood and adolescence [8]. In general, larger total brain, white matter, gray matter, cerebellum and hippocampal volumes have been reported to correlate with better IQ, memory and executive functioning in very preterm children and adolescents [9,10,26,33,34]. The current study not only confirmed these previous associations between brain size and general cognitive ability, but also found a novel relationship between amygdala volume and IQ. The amygdala is a structure in the limbic system involved in memory and emotional learning [35]. Smaller amygdala volume at age 8 years has been described in a preterm cohort of 25 children compared with controls, but no correlation between amygdala volume and IQ was noted [4].

In the current study, there was little evidence that prematurity affected the relationship between brain size and IQ. There have been reports of higher IQ scores with larger thalami and gray matter volume in typically developing children and adolescents aged 4-21 years, however none of those studies included preterm children [36-38].

The relationship between brain size and measures of basic educational skills has been less well studied than IQ, although math computation ability in preterm and term cohorts has been associated with reductions in specific gray matter regions [39,40]. In the current study we found evidence of a relationship between math computation and brain volume for most structures examined. Thus, our findings lend further support that smaller brain volumes may be neural markers of math computation difficulties.

Larger brain size was also associated with better literacy outcomes, although for both spelling and word reading the evidence for this relationship was weaker after adjustment for maternal education, social class and sex. Scott et al [41] also found that spelling was associated with regional gray matter volumes in very preterm and full-term 14-15 year olds, although the association was weaker in the very preterm group. While we found an association between larger brain volumes and higher literacy outcomes, this relationship did not differ as a function of prematurity.

While a previous study reported that total white matter and corpus callosum volume explained a large proportion of the variability in IQ within preterm adolescents [26], to our knowledge our study is the first to document that total brain tissue volume partially explains the differences between the EP and control groups on measures of IQ and basic educational skills. These findings suggest that brain volume is an important biomarker of cognitive and educational outcomes.

The current study has several strengths. It is the largest cohort of EP adolescents born in the 1990s(when surfactant was available) who have had MRI as well as detailed functional assessments. Being a regional cohort, the findings have important generalizability and provide invaluable information on long term outcomes for parents and staff caring for these EP infants. All participants were recruited prospectively, and the matched controls were recruited contemporaneously at birth. We not only measured IQ, but also assessed basic educational skills to gain a more comprehensive understanding of the functional consequences associated with brain size. In addition, the brain volumes were calculated using an automated system with manual editing to ensure optimal tracing. This method enabled more brain tissues and structures to be measured simultaneously compared with more laborious manual tracing methods.

We also acknowledge the limitations. The follow-up rates at age 18 were lower than follow-up at earlier ages, but given the complexity of the study, the duration of follow-up from birth, and the fact that families had moved to other countries or states of Australia over time and were inaccessible, the follow-up rates of 78% for the EP group and 61% for the controls are reasonable. When we compared characteristics of EP participants included in this study and those who were not, those without MRI data were more likely to have significant illness affecting their developmental outcomes, as well as higher rates of cerebral palsy. Some of these participants were too disabled to attempt any assessment, including the MRI. Thus, our results could have potentially demonstrated even larger differences between the EP and control groups if the entire cohort had been able to be assessed with MRI.

Examining brain volume is one of many ways to understand the neurological changes associated with preterm birth. Further work correlating other structural and functional information obtained from advanced MRI might also provide a more global understanding of changes related to extreme prematurity in adolescence.

In conclusion, EP adolescents born in the 1990s had smaller brain volumes than term-born controls. Not only are brain volumes important biomarkers of IQ and academic outcomes, but they appear to partially explain IQ and educational underperformance in adolescents born <28 weeks’ gestational age.
